# Mixed Infection with *cagA* Positive and *cagA* Negative Strains of *Helicobacter pylori* Lowers Disease Burden in The Gambia

**DOI:** 10.1371/journal.pone.0027954

**Published:** 2011-11-29

**Authors:** Ousman Secka, Martin Antonio, Douglas E. Berg, Mary Tapgun, Christian Bottomley, Vivat Thomas, Robert Walton, Tumani Corrah, Julian E. Thomas, Richard A. Adegbola

**Affiliations:** 1 Medical Research Council Unit, Fajara, The Gambia; 2 School of Clinical Medical Sciences, Newcastle University, Newcastle upon Tyne, United Kingdom; 3 Department of Molecular Microbiology, Washington University School of Medicine, St. Louis, Missouri, United States of America; 4 Medical Research Council, Tropical Epidemiology Group, London School of Hygiene and Tropical Medicine, London, United Kingdom; University of Osnabrueck, Germany

## Abstract

**Background:**

The prevalence of *Helicobacter pylori* including strains with putatively virulent genotypes is high, whereas the *H. pylori*-associated disease burden is low, in Africa compared to developed countries. In this study, we investigated the prevalence of virulence-related *H. pylori* genotypes and their association with gastroduodenal diseases in The Gambia.

**Methods and Findings:**

DNA extracted from biopsies and *H. pylori* cultures from 169 subjects with abdominal pain, dyspepsia or other gastroduodenal diseases were tested by PCR for *H. pylori*. The *H. pylori* positive samples were further tested for the *cagA* oncogene and *vacA* toxin gene.

One hundred and twenty one subjects (71.6%) were *H. pylori* positive. The *cagA* gene and more toxigenic *s1* and *m1* alleles of the *vacA* gene were found in 61.2%, 76.9% and 45.5% respectively of Gambian patients harbouring *H. pylori*. There was a high prevalence of *cagA* positive strains in patients with overt gastric diseases than those with non-ulcerative dyspepsia (NUD) (p = 0.05); however, mixed infection by *cagA* positive and *cagA* negative strains was more common in patients with NUD compared to patients with gastric disease (24.5% versus 0%; p = 0.002).

**Conclusion:**

This study shows that the prevalence of *H. pylor*i is high in dyspeptic patients in The Gambia and that many strains are of the putatively more virulent *cagA^+^, vacAs1* and *vacAm1* genotypes. This study has also shown significantly lower disease burden in Gambians infected with a mixture of *cag*-positive and *cag*-negative strains, relative to those containing only *cag*-positive or only *cag*-negative strains, which suggests that harbouring both *cag*-positive and *cag*-negative strains is protective.

## Introduction

*Helicobacter pylori* is a genetically diverse microaerophilic gram negative bacterial species that chronically infects the human gastric mucosa, often starting in infancy and lasting for life [1]. About 50% of the world's adult population is colonized, with prevalences of over 80% in many developing countries including The Gambia [2]–[4]. Earlier reports indicated high prevalence of *H. pylori* colonization, but a low frequency of *H. pylori*-associated disease in Africa [2], [5], [6], a phenomenon that was called the “African enigma” [2]. DNA sequencing of housekeeping and virulence genes has shown that different sets of genotypes predominate in different human populations [7]. Of particular interests have been *H. pylori's cagA* oncogene and toxigenic *s1* and *m1* alleles of its *vacA* gene, which have been implicated in gastroduodenal diseases caused by this pathogen both in epidemiologic [8], [9], experimental animal and cell culture infection [10] studies. This said, several studies from different world regions have not detected such an association [8], [11], [12], an outcome suggesting the possibility of other virulence-modulating factors.

Individuals can be colonized by either a single or multiple strains of *H. pylori*, and even colonization by what is initially a single strain can, over time, lead to the emergence of multiple *H. pylori* subpopulations, due variously to mutation or to genetic recombination either between duplicate sequences in the single strain's genome or with DNAs from other transiently colonizing strains [8]. The prevalence of such mixed infections has been reported to vary (5–68%) [13]–[17] depending on geographical region, whether in a developed or developing country (low and high overall infection risk, respectively), and probably also methods of analysis. The *H. pylori* virulence-associated vacuolating cytotoxin (*vacA*) and *cag* pathogenicity island (*cag* PAI) genes, and also the *cag* empty site in strains lacking the *cag* PAI, are typically found in only one copy per genome [17]–[20]. Accordingly, detection of both the *cagA* gene and the *cag* empty site, or of both *s1* and *s2* (signal sequence; at 5′ end of gene) or both *m1* and *m2* (middle region) alleles of *vacA* in a biopsy or in pool of *H. pylori* from a person indicates mixed infection.

We wondered if having mixed infection might influence the risk of gastric disease; for example, if strains of different genotypes might occupy a broader range of niches in the stomach as has been seen during experimental infection [21] and thereby impact on clinical outcome. In this study, we investigated the genotypes of *H. pylori* in The Gambia and the relation of apparently single versus mixed infections to gastroduodenal diseases.

## Materials and Methods

### Ethics statement

Ethical approval of this study was obtained from the joint Medical Research Council (MRC) Unit, The Gambia/Gambia Government Ethics Committee and Division of Microbiology Infectious Diseases (DMID) International Review Board.

### Patients

Clinical data from the MRC Unit in The Gambia revealed that of 428 patients with gastric complaints investigated by gastric endoscopy between 2003–2008, 8 (1.9%) had gastric carcinoma, 20 (4.7%) and 15 (3.5%) had gastric and duodenal ulcers respectively, and that the others (89.9%) did not have such overt disease (diagnosed as non-ulcer dyspepsia; NUD (data not shown). All patients referred for endoscopy to the MRC Unit during the years 2003 to 2008 were initially considered eligible for inclusion: All 169 subjects who agreed to join this study provided written informed consent; in addition, for children less than 18 years, antral biopsies were obtained only after informed written parental consent. Patients with severe oesophago-gastroduodenal disease, including those with gastro-oesophageal varices, a small number with advanced gastric cancer and those on *H. pylori* eradication therapy, were excluded from the study. One hundred and twenty one patients from whose biopsies we successfully amplified virulence genes were analysed here. The mean age of these subjects was 35, ranging from 9 to 80 years. All the subjects were Gambians and most of them (75) came from the Greater Banjul Area, 38 from the West Coast region, 5 from Lower River region and 3 from North Bank region of The Gambia.

### Gastroscopy results

Endoscopic examination showed that of the 121 study subjects, 11 had gastric ulcer, 7 had duodenal ulcer, 1 had both gastric and duodenal ulcers, 7 had gastric erosions, 1 had gastric carcinoma and all other subjects (94) who presented with either abdominal pain or dyspepsia had no evidence or history of gastric or duodenal ulcers.

The biopsies collected from patients were stored in Brain Heart Infusion (BHI) broth containing 20% glycerol and transported on ice to the laboratory for processing or stored at −70°C until used. Our previous data demonstrated that in The Gambia detection of mixed *H. pylori* strains in individual biopsies was best undertaken by PCR amplification directly from biopsy material rather than by bacterial culture [22].

### Genomic DNA extraction directly from biopsies

Total genomic DNA was extracted directly from the biopsy material by using a combination of bead-beater and the QIAamp DNA isolation kit (Qiagen, UK) as previously described [22].

### PCR amplification

PCR was performed to detect the *cagA* gene and *cag* empty site, and the signal sequence (*s1* and *s2*) and middle region (*m1* and *m2*) alleles of the *vacA* gene, as previously described [23] using the primers listed in table 1 and the following cycling conditions: 30 cycles of 94°C for 1 min, 55°C or 60°C for 1 min and 72°C for 1 min. The amplified genes were detected by electrophoresis in a 1.5% gel with ethidium bromide (500 ng/ml) and bands were visualized using Gel Doc 2000 (Bio-Rad laboratories, Milan, Italy). The presence of a particular gene or allele was inferred when a product of the expected size (table 1) was obtained using appropriate primers.

**Table 1:** Primers used in this study.

Region	Primer	Nucleotide sequence	bp	reference
*cagA*	cagA-FcagA-R	gat aac agg caa gct ttt gag g ctg caa aag att gtt tgg cag a	349	[23]
*cag* empty site	Luni-1R5280	aca ttt tgg cta aat aaa cgc tg ggt tgc acg cat ttt ccc tta atc	535	[23]
*vacAs1* & *vacAs2*	Va1-FVa1-R	atg gaa ata caa caa aça cac ctg ctt gaa tgc gcc aaa c	*s1* 259*s2* 289	[23]
*vacAm1*	Va3-FVa3-R	ggt caa aat gcg gtc atg g cca ttg gta cct gta gaa ac	290	[23]
*vacAm2*	Va4-FVa4-R	gga gcc cca gga aac att g cat aac tag cgc ctt gca c	352	[23]

### Statistical analysis

We assessed the prevalences of infection with single vs. multiple strains. For the *cagA* gene, for example, we noted the occurrence of *cagA* positive, *cag* empty site and mixed (*cagA* positive and *cag* PAI negative) infections. Prevalences were compared between disease groups and p-values were determined using Fisher's exact test.

## Results

### Prevalence of *H. pylori* genotypes

One hundred and twenty one patients of the 169 study participants were inferred to be infected with *H. pylori* when DNAs extracted from their biopsies were tested by PCR for the presence of *H. pylor*i *cagA* gene and *cag* empty site. Seventy four biopsies (61.2%) were positive for the *cagA* gene only, 21 (17.4%) were positive for the *cag* empty site only and 23 (19%) were positive for both. In parallel we also tested for the *vacA* gene presence and allele types. In all, 93 of 121 (76.9%) were positive only for the *vacAs1* allele, 23 (19.0%) were positive only for the *vacAs2* allele and 1 (0.8%) was positive for both. Only *m1* or only *m2* alleles of *vacA* were detected in 55 (45.5%) and 36 (29.8%) of biopsies tested respectively; both *m1* and *m2* (mixed infections) were found in 22 (18.2%) biopsies and up to 6.6% of biopsy DNAs failed to amplify for individual alleles (table 2).

**Table 2:** Prevalence of *Helicobacter pylori* genotypes.

*H. pylori* genotypes	n	%
*cagA^+^*	74	61.2
*cagA^−^*	21	17.4
*cagA^+^* & *cagA^−^*	23	19.0
No amplification of *cagA o*r *cag* emptysite	3	2.5
*s1*	93	76.9
*s2*	23	19.0
*s1* & *s2*	1	0.8
no amplification of *s1* or *s2*	4	3.3
*m1*	55	45.5
*m2*	36	29.8
*m1* & *m2*	22	18.2
No amplification of *m1* or *m2*	8	6.6

### Association between *H. pylori* genotypes

Of the 93 *H. pylori* strains that were positive only for *vacAs1*, 72 (77.4%) were *cagA* positive compared with only 1 (4.3%) *cagA* positive among the 23 strains that were positive only for *vacAs2*; most (16) of them contained the *cag* empty site allele only (table 3). Similarly, nearly all *s1m1* positive biopsies (92.5%) contained *cagA* genes, whereas none of those containing only *vacA s2m2* allele were *cagA* positive (table 3).

**Table 3:** Association of *vacA* with *cagA Helicobacter pylori* genotypes.

	*cagA* ^+^		c*agA* ^−^		*cagA* ^+^ & *cagA* ^−^		Incomplete *cagA*		
*H. pylori* genotypes	n	(%)	n	(%)	n	(%)	n	(%)	Total
*s1m1*	49	(92.5)	1	(1.9)	3	(5.7)	0	(0)	53
*s1m2*	9	(50)	3	(16.7)	5	(27.8)	1	(5.6)	18
*s2m2*	0	(0)	16	(88.9)	2	(11.1)	0	(0)	18
*s2m1*	0	(0)	0	(0)	0	(0)	0	(0)	0
*s1m1m2*	12	(66.7)	0	(0)	6	(33.3)	0	(0)	18
*s1s2m1m2*	0	(0)	0	(0)	1	(100)	0	(0)	1
*s2m1m2*	0	(0)	0	(0)	3	(100)	0	(0)	3
Incomplete *VacA*	4	(40)	1	(10)	3	(30)	2	(20)	10

Incomplete *cagA* = *cagA* and *cag* empty site were not detected.

Incomplete *vacA* = either *vacA s* or *vacA m* regions were not detected (4/10 *vacAs1* was detected & *vacAm* was missing, 2/10 *vacAs2* detected and *vacAm* missing, 2/10 *vacAm1* detected and *vacAs* missing and for 2/10 both *vacAs* and *vacAm* were missing).

### Association of *H. pylori* virulence genes with upper gastric diseases

*cagA* positive *H. pylor*i strains were found more frequently among study participants with gastroduodenal diseases than those with NUD: duodenal ulcers (6/7; 85.7%), gastric erosions (5/7, 71.4%), gastric ulcers (8/11, 72.7%); no overt gastric disease (53/94, 56.4%) (table 4). In the 27 patients with overt gastric disease, 77.8% were *cagA* positive compared to 56.4% of those with NUD (p-value = 0.05, table 5). Toxigenic *s1m1* alleles were found in 6 of the 11 (54.5%) patients diagnosed with gastric ulcer, 42.9%, 42.9% and 42.6% in those with duodenal ulcers, gastric erosions and NUD, respectively (table 6). The prevalence of *vacA* alleles were similar in the two groups of patients; overt disease vs. NUD. That is, no association was found between *vacA* alleles and clinical outcome (p = 0.94, table 7).

**Table 4:** Association between *cagA* genotypes and disease type.

	DU		GC		GE		GU		GUDU		NUD		Total	
*cagA* status	n	(%)	n	(%)	n	(%)	n	(%)	n	(%)	n	(%)	n	(%)
*cagA* ^+^	6	(85.7)	1	(100)	5	(71.4)	8	(72.7)	1	(100)	53	(56.4)	74	(61.2)
*cagA^−^*	1	(14.3)	0	(0)	2	(28.6)	3	(27.3)	0	(0)	15	(16.0)	21	(17.4)
*cagA^+^& cagA^−^*	0	(0)	0	(0)	0	(0)	0	(0)	0	(0)	23	(24.5)	23	(19.0)
No amplification	0	(0)	0	(0)	0	(0)	0	(0)	0	(0)	3	(3.2)	3	(2.5)
Total	7	(5.8)	1	(0.8)	7	(5.8)	11	(9.1)	1	(0.8)	94	(77.7)	121	(100)

DU = duodenal ulcer, GC = gastric carcinoma, GE = gastric erosion, GU = gastric ulcer, GUDU = gastric ulcer and duodenal ulcer, NUD = Non-ulcerative disease.

**Table 5:** *cagA* and clinical outcome.

	Overt gastric disease		NUD	
*cagA* status	n	%	n	%
*cagA^+^*	21	77.8	53	56.4
*cagA^−^*	6	22.2	15	16.0
*cagA^+^* & *cagA* ^−^	0	0	23	24.5
no amplification	0	0	3	3.2
Total	27	100	94	100

**Table 6:** Association between *vacA* genotypes and disease type.

*vacA status*	DU*		GC*		GE*		GU*		GUDU*		NUD*		Total	
	n	(%)	n	(%)	n	(%)	n	(%)	n	(%)	n	(%)	n	(%)
*s1m1*	3	(42.9)	1	(100)	3	(42.9)	6	(54.5)	0	(0)	40	(42.6)	53	(43.8)
*s1m2*	1	(14.3)	0	(0)	2	(28.6)	0	(0)	0	(0)	15	(16.0)	18	(14.9)
*s2m2*	0	(0)	0	(0)	2	(28.6)	3	(27.3)	0	(0)	13	(13.8)	18	(14.9)
*s1m1m2*	3	(42.9)	0	(0)	0	(0)	1	(9.1)	0	(0)	14	(14.9)	18	(14.9)
*s1s2m1m2*	0	(0)	0	(0)	0	(0)	0	(0)	0	(0)	1	(1.1)	1	(0.8)
*s2m1m2*	0	(0)	0	(0)	0	(0)	0	(0)	0	(0)	3	(3.2)	3	(2.5)
§Incomplete *vacA*	0	(0)	0	(0)	0	(0)	1	(9.1)	1	(100)	8	(8.5)	10	(8.3)
Total	7		1		7		11		1		94		121	

*Du = Duodenal ulcer; GC = gastric cancer; GE = gastric erosion; GU = gastric ulcer; GUDU = gastric and duodenal ulcers; NUD = non-ulcerative diseases.

§ Incomplete *vacA* = either *vacA s* or *vacA m* regions were not detected (4/10 *vacAs1* was detected & *vacAm* was missing, 2/10 *vacAs2* detected and *vacAm* missing, 2/10 *vacAm1* detected and *vacAs* missing and for 2/10 both *vacAs* and *vacAm* were missing).

**Table 7:** Association between *vacA* genotypes and clinical outcome.

	Overt gastric disease		NUD	
*H. pylori* genotypes	n	%	n	%
*s1m1*	13	48.1	40	42.5
*s1m2*	3	11.1	15	16.0
*s2m2*	5	18.5	13	13.8
*s1m1m2*	4	14.8	14	14.9
*s1s2m1m2*	0	0	1	1.1
*s2m1m2*	0	0	3	3.2
Incomplete *vacA*	2	7.4	8	8.5
Total	27	100	94	100

Incomplete *vacA* = either *vacA s* or *vacA m* regions were not detected (4/10 *vacAs1* was detected & *vacAm* was missing, 2/10 *vacAs2* detected and *vacAm* missing, 2/10 *vacAm1* detected and *vacAs* missing and for 2/10 both *vacAs* and *vacAm* were missing).

All 27 subjects with overt gastric diseases were of uniform *cagA* status (that is, uniquely *cagA* gene positive or *cag* empty site positive), whereas only 72.3% (68/94) of NUD were of uniform status; the other 23 contained mixed (*cagA* positive, *cag* empty site positive) infections. Three other biopsy samples did not give *cagA* gene or *cag* empty site amplification (table 5). This association between uniform *cagA* status and overt disease was statistically significant (p = 0.002).

In terms of age distribution, no association was found between age and overt gastric disease (24.5%<30 years, 12.5% 30–40 years and 27.8%>40 years; p-value = 0.26, table 8), or frequency of mixed infection (15.1%<30 years, 18.8% 30–40 years, 25%>40 years; p-value = 0.46, table 9).

**Table 8:** Association between age and disease.

	Overt disease		NUD		
Age groups	n	(%)	n	(%)	Total
<30 years	13	(24.5)	40	(74.5)	53
30–40 years	4	(12.5)	28	(87.5)	32
>40years	10	(27.8)	26	(72.2)	36

p-value = 0.26.

**Table 9:** Association between age and mixed infection.

	Mixed *cagA* (*cagA* ^+^ & *cagA* ^−^)		Uniform *cagA* status (*cagA* ^+^ or *cagA* ^−^)		No amplification for *cagA* gene		
Age groups	n	(%)	n	(%)	n	(%)	Total
<30 years	8	(15.1)	44	(83.0)	1	(1.9)	53
30–40 years	6	(18.8)	25	(78.1)	1	(3.1)	32
>40years	9	(25.0)	26	(72.2)	1	(2.8)	36

p-value = 0.46.

## Discussion

*H. pylori* infection is common in dyspeptic adults in The Gambia [4], [22], as is typical of developing countries. The range of *H. pylori* genotypes implicated in overt gastroduodenal disease as opposed to benign colonization or possibly even beneficial carriage [9], [24] had not been extensively investigated in Sub–Saharan Africa. Here we studied the distribution of *H. pylori*'s main virulence genes, *cagA* and toxigenic alleles of *vacA*, in The Gambia, and their possible associations with disease outcome.

The prevalence of gastroduodenal disease (10%) that we detected endoscopically is similar to that reported elsewhere in Sub-Saharan Africa [25] and may be lower than that in Europe and North America. However, these estimated prevalences should be interpreted with caution as they may not be representative of the general population, but instead indicate the prevalence of disease among people with gastric complaints of sufficient severity to prompt diagnostic endoscopy. We found that just over half of Gambian isolates (61.2%) carried the *cagA* gene (table 2) and that mixed infections (both *cagA* positive and *cag* PAI negative) were common. We also found toxigenic *vacAs1* and *m1* alleles to be abundant, but not universal (76.9% and 45.5%). In contrast, most *H. pylor*i strains from Egypt carried *vacAs2* and *m2* alleles, (57.1% and 85.7%, respectively) [9], whereas some 90% or more of strains in Japan, coastal China, and India carried *vacAs1* alleles [23], [26]–[28]. *vacAm1* type alleles were also nearly universal in Japanese main island strains, whereas *vacAm2* strains were predominant in coastal China [28], [29]. In this study, most of the *vacAs1* strains (77.4%) were *cagA* positive similar to what was observed in a study in South Africa [30]. Up to 6.6% of samples in this study failed to amplify for individual genes consistent with other findings [22], [30], perhaps due to PCR inhibitors or potent nucleases in some gastric biopsies as also suggested previously [22], [30], [31].

Thus, our data reinforce conclusions that different *H. pylori* genotypes, especially types of genes or alleles implicated in virulent vs. benign infections, predominate in different human populations. Strains of the *vacA s1m2* type were most common in coastal China, and also seemed to predominate in Southern Nigeria [32]; and both *s1m2* and *s2m2* allele types were abundant in South Africa [30]. This contrasts with the predominance of *vacA s1m1* strains found in our Gambian study participants. This apparent difference between Nigerian and Gambian strain genotypes could have several explanations, including the distance separating these two West African countries (>3000 km), or climatic differences (high rainfall and humidity in Southern Nigeria vs aridity for much of the year in The Gambia). Such explanations would entail genetic divergence by random genetic drift and selection for adaptation to local conditions respectively.

Previous reports of a lower than expected prevalence of *H. pylori* associated disease in Africa [2], [5], [6] might in principle reflect the influence of bacterial and/or human genotypes, environment including other infections (e.g. parasitic infections that affect host response to *H. pylori*), normal gut flora, diet (including anti-oxidants, salt, spices) or likelihood of seriously ill persons being diagnosed and their cases entered in registries. In accord with this last explanation, have been suggestions that gastroduodenal disease is actually common in Africa, that there is no African enigma [11], [12]. The *cagA* gene and *vacAs1* and *m1* alleles are often linked to severe disease, and the *vacAs2* and *m2* alleles with more benign infections (gastritis only) in other populations. This is partially reflected in our results: with respect to *cagA* we did not find a “Gambian-*H. pylori* virulence gene” enigma; disease associations with *vacA* were less clear cut. The possible effects of bacterial or human genetic and physiologic differences, food, history of other infections and other environmental and lifestyle factors, on outcomes of chronic *H. pylori* infections in sub-Saharan Africa merit further more detailed analyses.

Most important, was our finding that co-existence of *cagA* positive and *cagA* negative strains was significantly more common amongst patients with NUD than among those with overt disease, which suggests that mixed colonization is protective. In principle, protection against development of overt gastric disease might stem from simple competition – whereby carriage of an avirulent (*cagA*-negative) strain diminishes the vigour of growth of a coexisting virulent strain, thereby reducing its impact on host tissues. It is also possible that factors in *cag*-negative strains that diminish the impact of virulence proteins such as CagA might predominate during *cagA*-positive and *cagA*-negative mixed infections [33]. Or, more generally, an increased complexity of immune responses during chronic infection by multiple divergent *H. pylori* strains might effectively diminish the inflammatory action of an individual virulent strain, and thereby resultant pathology in host tissues, as noted with other infections [34]–[37]. In accord with this idea, the risk of developing overt disease seemed higher in subjects apparently colonized only with *cagA* negative *H. pylori*, than in those with mixed *cagA* positive and negative strains. Conversely, however, the presence of mixed infections might also stem from increased intrinsic host susceptibility to *H. pylori* infection and equally the development of a more severe clinical outcome [38], [39].

This study has revealed frequent gastro-duodenal disease among Gambians with gastric complaints. Many strains carried *cagA^+^* and *s1*, *m1* alleles of *vacA*, which are disease associated in many European and North American populations. Although *cagA* status was associated with disease in The Gambia, alleles of *vacA* were not. Comparison of our data with those from southern Nigeria pointed to a potentially significant difference in linkage of signal sequence (*s1* vs. *s2*) and middle region (*m1* vs. *m2*) alleles, which control the potency and tissue specificity of toxin action respectively (*s1m1* most common in The Gambia, vs. *s1m2* most common in Nigeria). The possibility that such differences reflect selection for optimal genotypes or random genetic drift in these well-separated West African nations merit further study. We suggest that our most interesting finding is the significantly lower disease burden in Gambians infected with a mixture of *cag*-positive and *cag*-negative strains, relative to those containing only *cag*-positive or only *cag*-negative strains. The possibility that repeated exposure would be beneficial in Sub-Saharan Africa and in developing countries more generally needs to be considered when developing more effective treatment strategies for treating *H. pylori* infection and risks of gastroduodenal disease [40].
